# Metropolitan Society of General Practitioners

**Published:** 1830-07-01

**Authors:** 


### PROSPECTUS OF THE "METROPOLITAN SOCIETY OF GENERAL PRACTITIONERS IN MEDICINE AND SURGERY."

GENERAL PRACTITIONERS.

A SOCIETY has been formed, which is entitled, "THE METROPOLITAN SOCIETY OF GENERAL PRACTITIONERS IN MEDICINE AND SURGERY," and is intended as an union of the Practitioners of this class throughout England and Wales, for the protection of their mutual and individual interests; having the following objects in view:—

1st. Such alteration of existing laws and customs as shall promote the prosperity and respectability of the general body of Practitioners.

2nd. The adoption of such measures as may be conducive to the advancement of medical science and of professional information.

3rd. The periodical assemblage of the members for literary and scientific discussion—for the cultivation of social intercourse, and for the consideration of general measures relative to the Society.

4th. The creation of a fund to be appropriated to the protection of the Members, and for the general exigencies of the Society.

5th. The establishment of a *benevolent* fund, by contributions from Members of the Profession at large and other charitable persons, for the relief of distressed medical men and their families.

The limits of a prospectus will not allow of a full detail of the objects contemplated; but it may be observed, in addition to the foregoing general statement, that it is intended, as soon as practicable, to effect some regulation respecting the mode of professional compensation; and, if necessary, to procure a legislative enactment to authorise the General Practitioner to make a fair and open charge for his services. It is also intended to protect individually those Members who may become involved in questions which may be considered by the Committee to affect the interests of the Society as a body.

Notwithstanding that there are numerous charitable funds for relieving distressed members of particular branches of the Medical profession, it is found that there are many Members of that Profession who are not objects of relief from any of those funds; and it is, therefore, to supply this desideratum, that the Plan of a General Benevolent Fund has been adopted, the application of which, it is intended, *should not be confined to this Society exclusively*, but should be extended, at the discretion of the Committee, *to every Member of the Profession.*

The affairs of the Society are under the management of a President, Vice-President, and a Committee.

A House, or Chambers, will be engaged, as early as possible, for the use of the Society.

The Society will meet at such stated periods, and in such manner, as will be hereafter determined.

The foregoing is a brief statement of the views of the Founders of this Society, and of the advantages intended from its institution, the Plan of which may be enlarged, or curtailed, according to the support it may receive.

The Committee of Management entertain a confident hope that the Society will be of great utility to the general body of Practitioners, whose attention to this subject is earnestly recommended.

WILLIAM GAITSKELL, *President.*

*It is requested that all applications and communications be made and addressed (post paid) to* Mr. W. SENHOUSE GAITSKELL, *Solicitor,* 21, *Stamford Street, Blackfriars.*

---

